# Simian immunodeficiency virus (SIV) envelope quasispecies transmission and evolution in infant rhesus macaques after oral challenge with uncloned SIVmac251: increased diversity is associated with neutralizing antibodies and improved survival in previously immunized animals

**DOI:** 10.1186/1743-422X-2-11

**Published:** 2005-02-14

**Authors:** Jennifer L Greenier, Koen KA Van Rompay, David Montefiori, Patricia Earl, Bernard Moss, Marta L Marthas

**Affiliations:** 1 California National Primate Research Center, University of California, Davis, CA 95616, USA; 2 Duke University Medical Center, Durham, NC 27710, USA; 3 Laboratory of Viral Diseases, National Institutes of Health, Bethesda, MD 20892, USA; 4 Department of Pathology, Microbiology and Immunology, School of Veterinary Medicine, University of California, Davis, CA 95616, USA

**Keywords:** pediatric, vaccine, HIV, HMA

## Abstract

**Background:**

Oral infection of infant macaques with simian immunodeficiency virus (SIV) is a useful animal model to test interventions to reduce postnatal HIV transmission via breast-feeding. We previously demonstrated that immunization of infant rhesus macaques with either modified vaccinia virus Ankara (MVA) expressing SIV Gag, Pol and Env, or live-attenuated SIVmac1A11 resulted in lower viremia and longer survival compared to unimmunized controls after oral challenge with virulent SIVmac251 (Van Rompay *et al.*, J. Virology 77:179–190, 2003). Here we evaluate the impact of these vaccines on oral transmission and evolution of SIV envelope variants.

**Results:**

Limiting dilution analysis of SIV RNA followed by heteroduplex mobility assays of the V1–V2 envelope (*env*) region revealed two major *env *variants in the uncloned SIVmac251 inoculum. Plasma sampled from all infants 1 week after challenge contained heterogeneous SIV *env *populations including one or both of the most common *env *variants in the virus inoculum; no consistent differences in patterns of *env *variants were found between vaccinated and unvaccinated infants. However, SIV *env *variant populations diverged in most vaccinated monkeys 3 to 5 months after challenge, in association with the development of neutralizing antibodies.

**Conclusions:**

These patterns of viral envelope diversity, immune responses and disease course in SIV-infected infant macaques are similar to observations in HIV-infected children, and underscore the relevance of this pediatric animal model. The results also support the concept that neonatal immunization with HIV vaccines might modulate disease progression in infants infected with HIV by breast-feeding.

## Background

The continued need for breast-feeding in developing countries due to nutritional or socio-economic reasons poses a considerable risk for postnatal mother-to-child transmission of HIV, and breastfeeding is estimated to account for 33–50% of infant HIV infections worldwide [1-5]. This dilemma underscores the need for a vaccine that, when administered shortly after birth to the infant, could protect against HIV transmission via breast-feeding. The ultimate goal of a neonatal HIV vaccine is to prevent infection; however, vaccination of newborns of HIV-infected women early in life may elicit HIV-specific immune responses that substantially reduce infant disease progression in the event that breast milk transmission occurs.

Advances in the understanding of the mechanisms of oral transmission of HIV variants may aid the development of an effective infant HIV-1 vaccine. Recent studies have demonstrated that infants of HIV-infected women can be infected with single or multiple HIV variants [6,7] shortly before or during the birth process. However, little is known regarding the diversity of HIV transmitted by breastfeeding. These questions are difficult to address in human studies because the characteristics of HIV variants in breast-milk at the time of transmission are unknown. In addition, it is often difficult to obtain virus from infants at early times after HIV infection. Finally, the presence in infants of different levels of transplacentally transferred HIV-specific maternal antibodies with differing anti-viral properties complicates assessments of HIV variant transmission.

Longitudinal studies of HIV-infected adults have shown that the rate of disease progression is inversely related to the rate of evolution of HIV envelope quasispecies [8,9]. Also, without antiviral treatment, virus-specific immune responses are directly related to HIV quasispecies evolution [10]. The reported relationship between HIV envelope variant evolution and disease progression in HIV-infected infants and children is contradictory. Some studies have found greater HIV envelope variant evolution in rapid progressors [11-13] while other investigations have found that slowly progressing HIV-infected children have greater HIV quasispecies divergence or diversity over time [14,15]. However, all of these retrospective studies necessarily evaluated HIV variant evolution in a limited number of serial blood samples during the first months of life from a small number of HIV-infected children (two to six per cohort). More recently, a longitudinal study of 10 perinatally HIV-infected children found that changes in HIV envelope quasispecies during the first year of life were associated with a better clinical outcome [7]. A few reports have described a correlation between nascent HIV-specific immune responses, the evolution of HIV variants and disease progression in HIV-infected infants [16,17].

Simian immunodeficiency virus (SIV) infection of infant macaques is a useful and relevant animal model of pediatric HIV infection for rapidly testing the efficacy of pediatric HIV vaccine and drug interventions [18-20]. This SIV/infant macaque model was previously used to assess the efficacy of two vaccines, (i) modified vaccinia virus Ankara (MVA) expressing SIV Gag, Pol and Env (MVA-SIVgpe) and (ii) live-attenuated SIVmac1A11, against oral challenge with virulent uncloned SIVmac251. We reported an improved clinical outcome (i.e., disease-free survival) for vaccinated compared with unvaccinated infants, which was associated with reduced plasma SIV RNA and sustained SIV-specific humoral immune responses [21]. Here in this report, we used a heteroduplex mobility assay (HMA) to evaluate the genetic diversity in the V1–V2 envelope (*env*) region of SIV variants present in the SIVmac251 virus inoculum and compare the transmission and evolution of the SIV *env *quasispecies in plasma following oral inoculation of these vaccinated and unvaccinated infant macaques. Three major questions were addressed: (i) Compared to the SIVmac251 virus inoculum, are few SIV envelope variants transmitted orally?, (ii) Is the lower viremia and better clinical outcome of vaccinated infants related to the initial genetic diversity of SIV *env *quasispecies?, and, (iii) Is the evolution of SIV envelope quasispecies during the course of infection associated with the development of SIV neutralizing antibody? We demonstrate that while the vaccines did not modulate oral transmission of viral variants, an association was found between vaccine-induced enhanced antiviral immune responses, increased *env *diversity, and a slower disease course. These findings in vaccinated infant macaques are similar to observations in HIV-infected children with slow disease progression and support the relevance of the SIV infant macaque model for developing neonatal vaccine strategies to prevent pediatric HIV infection and AIDS.

## Results

### Characterization of variants in SIVmac251-5/98 virus stock

**Figure 1:**
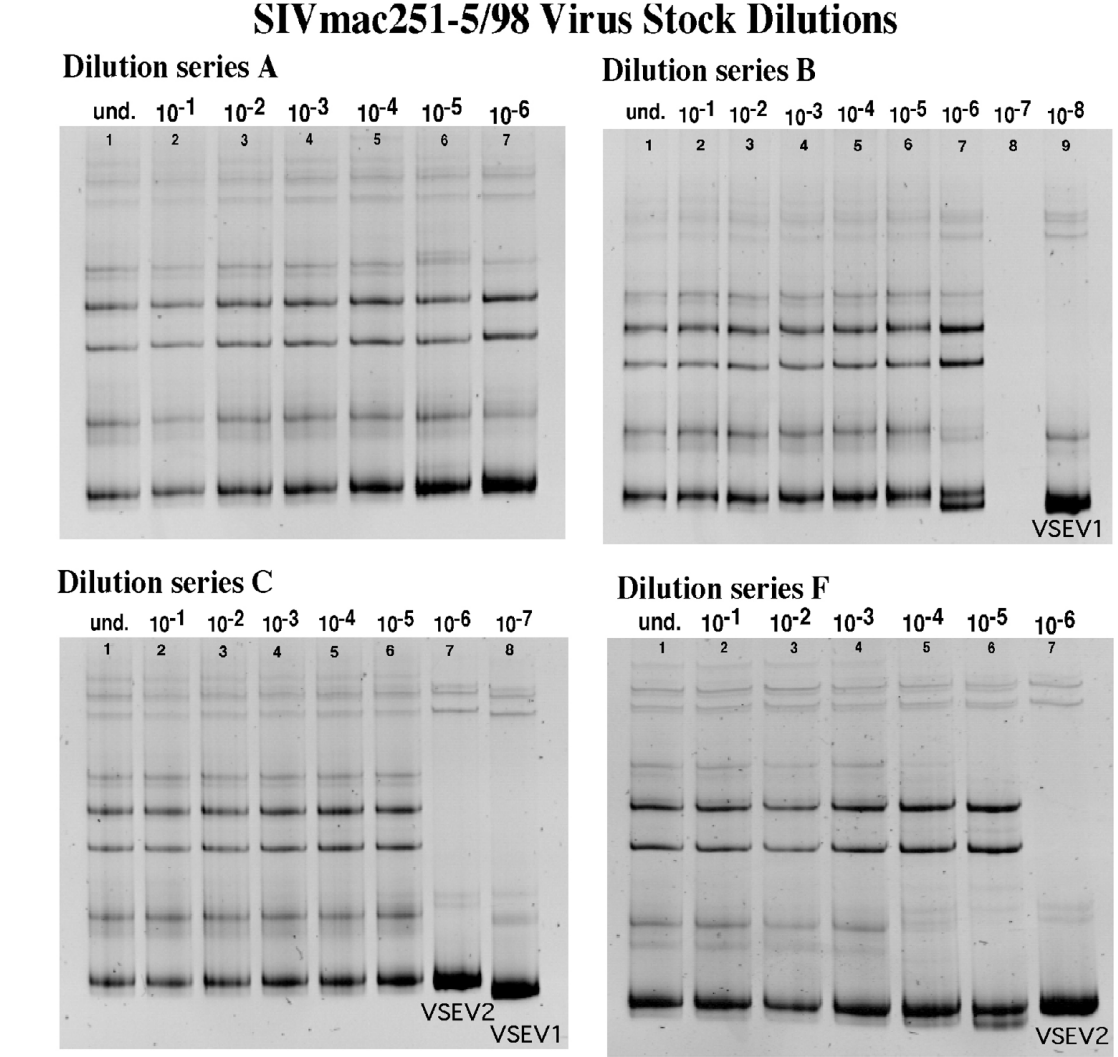
**Characterization of variants in SIVmac251-5/98 virus stock. **HMA analysis of four separate dilution series of viral RNA from the SIVmac251-5/98 virus stock is shown. The presence of multiple bands in the undiluted samples (lane 1 of each gel) reveals the virus stock was comprised of a diverse viral population. The last lane of each gel shows the variants in the highest dilution that yielded an RT-PCR product. Dilution series A shows an example of a dilution experiment that did not result in a virus stock endpoint (homogenous variant population); the 10^-6 ^dilution included more than 1 variant, while the next dilutions (10^-7^–10^-9^) dilution did not yield RT-PCR products, and therefore no variant pattern is shown for those dilutions. This dilution pattern was observed in 3 of 6 dilution series (other 2 not shown). For the other 3 dilution series (B, C, and F), the variant (band) remaining in the highest dilution was considered to be the most common variant, and was designated the Virus Stock Endpoint Variant (VSEV). Dilution series B: no product was amplified from the 10^-7 ^dilution (lane 8), but a product was amplified from the 10^-8 ^dilution (lane 9). Dilution series C: lanes 7 and 8 show the presence of 2 different variants (VSEV-1 and VSEV-2) in the endpoint dilutions (10^-6 ^and 10^-7^) of this series. Dilution series F; the 10^-6 ^dilution in this series harbored an endpoint variant that migrated to the same gel position as VSEV-2 in dilution series C.

HMA analysis revealed that the undiluted SIVmac251-5/98 virus stock was comprised of a diverse population of V1–V2 *env *variants. To determine the most common variant(s) in the virus stock, six independent serial dilution experiments were conducted. Viral RNA was isolated from 1 ml of virus stock and 10-fold dilution series (undiluted to 10^-9^) of the RNA were prepared from 6 separate aliquots of virus stock. The resulting RNA was analyzed by RT-PCR and HMA. Figure 1 shows the results of 4 of these 6 separate virus stock dilution/HMA experiments. The observation that multiple heteroduplex bands were observed through the 10^-5 ^or 10^-6 ^dilutions of viral RNA indicates that the undiluted SIVmac251-5/98 stock contains multiple *env *variants at high frequency. An RT-PCR endpoint (i.e. dilution to a single variant) was not reached in 3 of the 6 dilution experiments. An example of this is shown in dilution series A (Figure 1). In the other 3 dilution series (Fig. 1, series B, C, and F), the last dilution that yielded an RT-PCR product consisted of a homogeneous population of envelope variants represented by one main variant (homoduplex band). This endpoint variant was designated the virus stock endpoint variant (VSEV). The fact that endpoint variants were reached at different dilutions for each dilution series is probably due to the variability at each step of these independently performed experiments.

**Figure 2:**
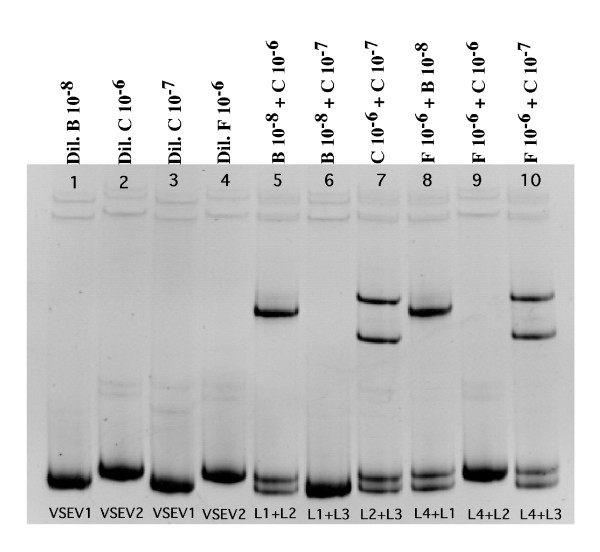
**Characterization of the dominant variants in SIVmac251-5/98 virus stock. **HMA analysis of all four endpoint variants shown in Fig. 1 (lanes 1–4) and all possible pairwise mixtures of those variants (lanes 5-10) are shown. Letters B, C, and F refer to the dilution series shown in Fig. 1. Lane numbers refer to the lane designations of the variants that were mixed in lanes 5–10 (e.g., L1 + L2 indicates that the variants shown in lanes 1 and 2 were mixed). Lane 6 shows that the 2 endpoint variants labeled VSEV-1 (B 10^-8 ^and C 10^-7^) are similar variants due to the formation of a single homoduplex and no heteroduplexes when these 2 variants were mixed. Lane 9 indicates that the 2 endpoint variants labeled VSEV-2 (C 10^-6 ^and F 10^-6^) in Fig. 1 are very similar. The formation of heteroduplexes and two main homoduplexes in the mixtures shown in lanes 5, 7, 8, and 10 indicate that VSEV-1 and VSEV-2 do not share the same V1–V2 envelope sequence.

The VSEV in dilution series B and F had different mobilities on the HMA gel (Fig. 1). Dilution series C resulted in two endpoint variants, one at 10^-6 ^and the other at 10^-7^; the positions of these two VSEV corresponded to one of each of the two VSEV in dilution series B and F. Thus, the dilution of the virus stock to an RT-PCR endpoint resulted in 4 independent variants (represented by homoduplex bands) that migrated to two different positions on the HMA gels. Based on these positions, the homoduplex bands that migrated furthest were referred to as VSEV-1 and the variants that migrated a shorter distance were designated VSEV-2 (Fig. 1). To confirm that the four endpoint homoduplexes represented only two variants, an HMA mixture experiment was performed, in which all pairwise combinations of the virus stock endpoint variants were mixed prior to HMA [22]. These experiments demonstrated that the two variants designated VSEV-1 are indeed similar (i.e., = 1–2% difference in nucleotides with no insertion/deletion), as their mixtures resulted in the formation of a single homoduplex band on an HMA gel; similarly, the two variants referred to as VSEV-2 are similar (Fig. 2). In contrast, the formation of heteroduplexes and two main homoduplexes in the mixtures of VSEV-1 and VSEV-2 demonstrate that these 2 variants are significantly different from each other (Fig. 2). Thus, VSEV-1 and VSEV-2 are 2 distinct variants that exist at similar frequencies and represent the most common variants in the undiluted SIVmac251-5/98 virus stock. These results are consistent with observations of the virus stock from which SIVmac251-5/98 was made [22].

### Experimental design of animal experiments and summary of outcome

Nineteen newborn rhesus macaques were divided into 5 experimental vaccine groups (table 1). Group 1 (n = 5) consisted of unimmunized control animals. Group 2 (n = 2), group 3 (n = 4) and group 4 (n = 4) were vaccinated with MVA-SIVgpe at 0 and 3 weeks of age; group 4 had maternally-derived SIV antibodies (due to immunization of their mothers with inactivated SIV). Group 5 (n = 4) was immunized with live-attenuated SIVmac1A11 at 0 and 3 weeks of age. As described elsewhere [21], except for group 2, all other groups were inoculated orally with SIVmac251-5/98 at 4 weeks of age; all these animals became persistently viremic, but the immunized animals had lower virus levels, enhanced antiviral immune responses and a delayed disease course in comparison to the unimmunized animals. Four of the 5 unimmunized infected animals developed AIDS within 14 weeks of age, while the fifth animal needed euthanasia at 28 weeks. Four MVA-SIVgpe-vaccinated SIVmac251-5/98-infected animals developed AIDS by 19 to 27 weeks of age (2 animals of groups 3 and 4 each; table 1). The remaining eight vaccinated SIVmac251-5/98-infected infants, including all four SIVmac1A11-vaccinated animals, were clinically stable at the end of the observation period (28 weeks of age).

**Table 1:** Experimental design and summary of outcome.

**Immunization^a ^groups and animal numbers**	**sex**	**MHC I alleles^b^**		**Variant Pattern^c^**	**Week 1 Plasma Viral RNA^d^**	**Time of euthanasia (wks)^e^**
					
		MamuA*01	MamuB*01			
***Group 1 Unvaccinated + SIVmac251***						
31319	M	+	+	A	4.3 × 10^7^	13
31321	M	+/-	-	A	1.7 × 10^8^	28
31322	F	+/-	+/-	A	1.2 × 10^8^	14
31325	M	+	+	B	5.5 × 10^6^	12
31608^f^	F	+/-	+/-	C	7.5 × 10^5^	11
***Group 2 MVA-SIVgpe only***						
31480	M	-	-	na	na	na
31488	M	+/-	+/-	na	na	na
***Group 3 MVA-SIVgpe + SIVmac251***						
31378	M	-	-	A	4.8 × 10^5^	28^g^
31533	M	+/-	-	A	3.7 × 10^7^	26
31540	M	+/-	-	C	2.5 × 10^7^	28^g^
31542	M	-	-	B	3.3 × 10^5^	26
***Group 4 MVA-SIVgpe with Mat. Abs. + SIVmac251***						
31526	M	+/-	+/-	A	6.9 × 10^7^	27
31732	F	-	+/-	A	1.8 × 10^7^	19
31833	F	+/-	-	A/C	4.5 × 10^5^	28^g^
31856	F	-	+/-	B	1.4 × 10^6^	28^g^
***Group 5 SIVmac1A11 + SIVmac251***						
31777	F	+/-	-	A	6.8 × 10^7^	28^g^
31778	F	-	-	B	4.7 × 10^5^	28^g^
31779	F	-	-	A	2.3 × 10^8^	28^g^
31780	F	+/-	-	A	9.9 × 10^7^	28^g^

^a ^Vaccine administered in 2 doses, at birth and 3 weeks of age. Animals of groups 1, 3, 4 and 5 were challenged orally at 4 weeks of age with SIVmac251-5/98.

^b ^The presence of the MHC type I alleles of MamuA*01 and MamuB*01 is indicated as + (present, but unknown whether homozygous or heterozygous), +/- (heterozygous based on known haplotypes of parents), and - (homozygous for absence of particular allele).

^c ^Variants in plasma at one week post-challenge with SIVmac251-5/98.

^d ^Copies of viral RNA per ml one week after challenge with SIVmac251-5/98, as measured by bDNA assay.

^e ^Age (weeks) at time of euthanasia.

^f ^Infant 31608 was born to an SIVmac251-infected macaque, and thus had maternal anti-SIV antibodies, but no virus was detected in this infant at 4 weeks of age.

^g ^indicates that animal was clinically stable at time of experimental euthanasia at 28 weeks of age; all other SIV-infected animals were euthanized due to life-threatening disease prior to or at 28 weeks of age. The animals of group 2 were not euthanized. na indicates not applicable.

### Detection of SIV envelope variants in plasma of neonates early after oral inoculation

**Figure 3:**
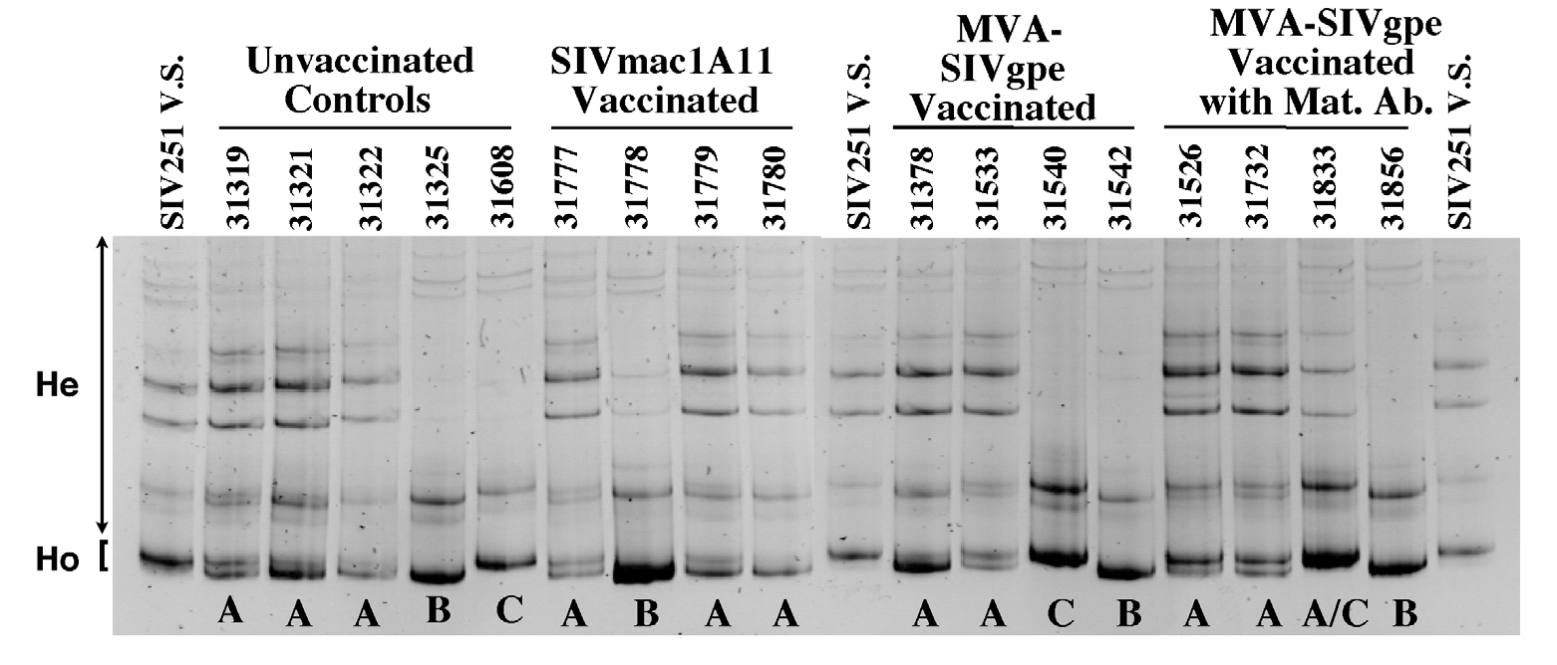
**Variant populations present in plasma of infant macaques one week after oral challenge with SIVmac251-5/98**. RT-PCR and HMA analysis was performed on replicate samples to confirm reproducibility of the results. Three main transmission patterns were observed, labeled A (multiple variants; diverse virus population), B and C (one major homoduplex (Ho) with a few faint heteroduplexes (He); relatively homogenous virus population). One infant (31833) harbored a plasma virus population that had elements of both transmission patterns A and C. SIV251 V.S. indicates the SIVmac251-5/98 virus stock.

The genetic diversity of SIV *env *variant populations in the plasma of the infant monkeys one week after oral inoculation with SIVmac251 was analyzed by HMA (Fig. 3). Each plasma sample was analyzed in replicates (≥ 2) to assure reproducibility of the gel banding patterns. As indicated by the presence of heteroduplex bands, all infants were infected with multiple SIV *env *variants, indicating that the SIVmac251-5/98 virus stock contained several variants capable of establishing infection by the oral route. However, there were differences in HMA banding patterns. In each group, some animals had several strong heteroduplex bands; this pattern of variant transmission was referred to as infection pattern A (e.g. Fig. 3, animal 31319). In contrast, one or two infants in each group were infected with a genetically more homogenous variant population, consisting of one major variant (homoduplex band), while heteroduplex bands were less pronounced. These monkeys infected with genetically more homogeneous viral populations harbored one of two main *env *variants, distinguished by different electrophoretic mobilities of the homoduplexes representing these variants. These more homogeneous variant populations were referred to as infection patterns B and C (e.g. Fig. 3, animals 31325 and 31608, respectively). Infection pattern C contained a homoduplex band that migrated slightly slower than the homoduplex band characterizing infection pattern B. One newborn in each vaccine group was infected with a SIV variant of transmission pattern B. Infection pattern C was detected in one newborn of each group except the SIVmac1A11 vaccinates (table 1, group 5). Therefore, no substantial difference was observed among the different vaccine groups in viral genetic diversity in plasma collected 1 week after virus inoculation. However, all but one infant (31540) infected with more homogenous populations of *env *variants (infection patterns B or C) had 10- to 100-fold lower virus levels one week after SIVmac251 challenge than all but one infant (31378) infected with more heterogeneous populations of SIV variants (transmission pattern A, Table 1). This association of homogeneous viral variants with reduced SIV RNA in plasma at 1 week after infection was statistically significant (P ≤ 0.05; one-sided Fisher's Exact test) but did not persist. From week 2 after challenge throughout the duration of the study, plasma SIV RNA levels showed no correlation with the initial SIV variant pattern detected in plasma. The rate of disease progression in these animals was also not associated with the initial envelope variant transmission patterns (table 1). Further, there was no correlation between the presence of the MHC type I alleles Mamu-A*01 or Mamu-B*01 and the viral variant infection patterns, levels of SIV RNA in plasma, or disease progression (table 1).

To determine which SIV envelope variant was present in the highest frequency in each infection pattern, serial end-point dilution experiments were performed with RNA isolated from plasma collected one week after SIVmac251 challenge, followed by RT-PCR and HMA. Similar to the methods described above, mixture experiments were then performed, including with VSEV-1 and VSEV-2. These experiments demonstrated that 1 week after infection, the most common variants in animals with the more homogenous transmission patterns B and C were similar (i.e., less than 1–2 % difference based on the absence of heteroduplex bands) to VSEV-1 and VSEV-2, respectively (data not shown). The most common variants by end-point dilution in the 11 monkeys with transmission pattern A and A/C were similar to VSEV-1 (5 animals), or VSEV-2 (5 animals) or both (1 animal).

### Greater quasispecies diversity in vaccinated compared to control infants during chronic SIV infection

**Figure 4:**
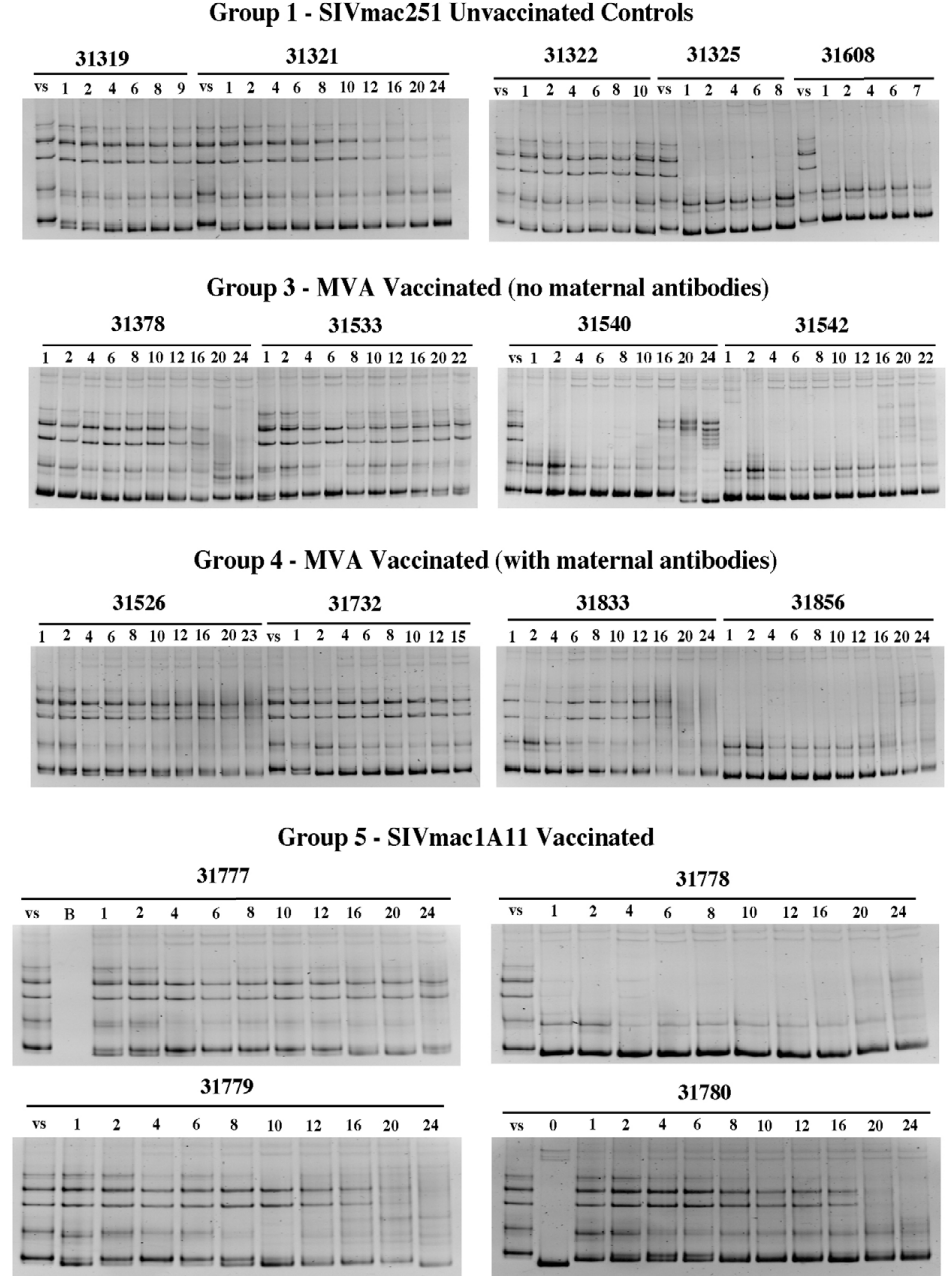
**Evolution of plasma variants in SIVmac251-5/98-infected infant macaques**. HMA analysis was performed on sequential plasma RNA samples, and each analysis was done at least twice to assure reproducibility. Virus diversification is evidenced by the detection of additional minor heteroduplex bands, the disappearance of major heteroduplex bands, and/or the decrease in density of the homoduplex bands. V.S. indicates the SIVmac251-5/98 virus stock. The lane numbers refer to the number of weeks after SIVmac251-5/98 inoculation (which was performed at 4 weeks of age). The homoduplex band for week 0 for animal 31780 (prior to SIVmac251 challenge) represents the vaccine virus SIVmac1A11; viral RNA levels for the other SIVmac1A11-immunized animals at this time were too low to result in a detectable RT-PCR product.

**Figure 5:**
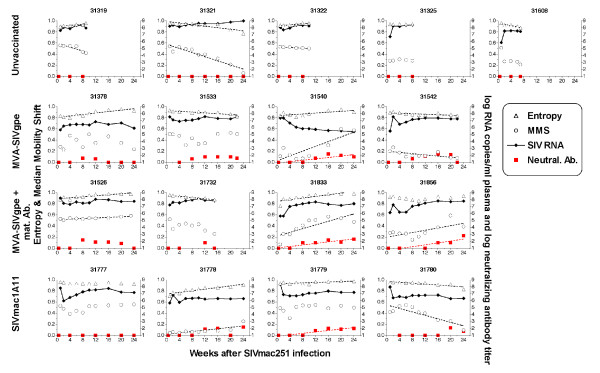
**Evolution of viral diversity and SIV neutralizing antibody response. **HMA data for each animal (Fig. 4) were further analyzed by calculating the entropy and the median mobility shift (MMS). Viral RNA levels were measured by bDNA. SIV neutralizing antibodies were determined as described in the Materials and Methods; neutralizing antibody titers below cut-off value (i.e., < 30) were given a value of 10 for presentation on these graphs. Dashed lines indicate a regression line for entropy, MMS or neutralizing antibody titer that is significantly different (P < 0.05) from zero (i.e. significantly increasing or decreasing values from 1 week to 24 weeks pc, with r^2 ^values ≥ 0.45).

HMA was used to analyze the evolution of genetic diversity of V1–V2 *env *populations in plasma of the monkeys during the course of infection (1 week after oral SIVmac251-5/98 challenge until euthanasia) (Fig. 4). Results from two standard measures of the nucleotide sequence heterogeneity of V1–V2 *env *plasma variants derived from the HMA analyses are shown in Fig. 5: (i) entropy (E), an estimate of the overall viral RNA sequence complexity for each sample and, (ii) median mobility shift (MMS), a measure of the SIV quasispecies sequence divergence reflected by the degree of base-pair mismatch after DNA strand re-annealing of strands of envelope variants [8].

The diversity of SIV *env *quasispecies in plasma varied among animals at the first sample (1 week after challenge) as indicated by the gel banding pattern (Fig. 4) and entropy measures (Fig. 5). Entropy for SIV *env *populations was high (> 0.9) for all unvaccinated animals (Group 1) and for 7 of the 12 vaccinated animals (Fig. 5). Initial entropy < 0.9 for vaccinated animals was associated with lower SIV RNA in plasma at 1 week after challenge (P < 0.05; Fisher's Exact Test). No consistent pattern of entropy over the 24 week course of infection was observed; in two of the five controls (31321, 31608) and three of the 12 vaccinates (31533, 31732, 31780) entropy decreased near the time of euthanasia. Overall, there was no association of SIV envelope diversity as measured by entropy with either viral RNA levels or virus-specific neutralizing antibodies in plasma (see below and Fig. 5).

The sequence divergence of SIV envelope variants in plasma of each animal over time was estimated by the MMS, shown in Fig. 5. Four of the five unvaccinated animals had initial MMS values ≥ 0.5 which decreased at varying rates until the time of euthanasia; the remaining control animal (31325) had an initial MMS < 0.3 which did not change significantly over the course of infection (Fig. 5). Thus, in unvaccinated infants the population of SIV env variants in plasma exhibited either no sequence divergence or increasing sequence similarity over time; this observation is consistent with the absence of sustained SIV-specific immune responses in these animals ([21]; see below). There was no association between MMS values and SIV RNA plasma levels for these unimmunized animals.

For 3 of the 4 vaccinated animals that developed AIDS within the observation period of 28 weeks (animals 31732, 31533, 31542), we also observed little change or a decrease of genetic divergence (i.e., as measured by stable or decreasing MMS values) of plasma *env *variant quasispecies. In contrast, diversification in plasma SIV *env *variant populations (i.e., a significant increase in MSS values) was observed by 3 to 5 months of infection in 4 of the 8 vaccinated monkeys (31540, 31833, 31856 and 31778) that were still relatively healthy at 28 weeks (Fig. 5). This diversification corresponded to the detection of different patterns of heteroduplex bands and/or fainter homoduplex bands over time (Fig. 4). Although increased diversification seemed to correlate with improved disease-free survival, this diversification was not associated with any obvious changes in plasma virus levels.

### SIV neutralizing antibodies in vaccinates correlate with evolution of SIV quasispecies diversity

The possibility that SIV envelope-specific immune responses were associated with the observed plasma SIV RNA levels or evolution of SIV *env *quasispecies was evaluated by measuring levels of plasma antibodies that neutralized the homologous challenge virus, SIVmac251-5/98, during the course of infection (Fig. 5). SIV neutralizing antibodies were detected in none of the unvaccinated control animals, but in all except one (31777) of the 12 vaccinated animals within 16 to 20 weeks after infection (Fig. 5). Although the presence of SIV neutralizing antibodies was associated with increased survival of the vaccinated animals, no obvious relationship was detected between the SIV neutralizing antibody levels and either SIV RNA plasma levels or entropy over time in vaccinated animals. However, in the 5 animals with increasing sequence divergence (i.e., increasing MMS values; animals 31540, 31526, 31833, 31856 and 31778), neutralizing antibodies were detected around the time that MMS values increased, and the neutralizing antibody response was sustained (i.e., detectable in ≥ 3 plasma samples) in these 5 animals (Fig. 5). In contrast, animals with stable or declining MMS values had sustained (31533, 31542, 31779), transiently detected (31378, 31732, 31780) or undetectable (31319, 31321, 31322, 31325, 31608, 31777) anti-SIV neutralizing antibodies. Thus, a sustained SIV neutralizing antibody response was associated with increased divergence of SIV envelope variants in plasma (P = 0.009; Fisher's Exact test).

## Discussion

The present study is among the most comprehensive longitudinal studies describing SIV envelope variation *in vivo *following mucosal SIV infection of infant macaques. In this study, we examined the extent of genetic diversity of the SIV envelope variant pool in the plasma of infant macaques that were inoculated orally at 4 weeks of age with an uncloned, genetically diverse virus stock SIVmac251-5/98. In addition, this is the first study to evaluate whether the transmission and evolution of viral variants was modulated by two different SIV vaccines, MVA-SIVgpe and SIVmac1A11, or the presence of maternally-derived anti-SIV antibodies.

HMA analysis revealed that the animals became infected with multiple SIV envelope variant populations, but which predominantly consisted of one of two single envelope variants that were very similar to the two most common variants in the SIVmac251-5/98 stock. These results are consistent with reports of mother-to-infant HIV transmission of multiple variants [23-25], single variants [14,26,27] or both [6,28-31], but inconsistent with studies reporting vertical transmission of single, minor variants [10,26,32-34] from the mothers' virus population. This discrepancy could be explained by differences in the HIV inoculum regarding dose, virulence and genetic diversity compared to SIV. In the present study, macaques were inoculated with a relatively high dose of SIVmac251-5/98, while infection of human infants is likely to occur due to exposure to lower amounts of virus. An inherent limitation of studies of vertical transmission of HIV is that the exact timing of infection is usually unknown, and therefore the mothers' population of viral variants at the time of transmission and the source (e.g., breast-milk) and dose of virus is unknown. Our observation that oral exposure of 17 infant macaques to the same dose of the same virus stock resulted in different transmission patterns further underscores the complexity of studying variant transmission in humans, and suggests that the different outcomes observed for vertical transmission of HIV may not necessarily reflect "selection" of HIV variants but may be more a stochastic event. In this context, studies looking at the effect of heterogeneity of viral variants in the HIV-1 infected mother and the rate of vertical transmission have also shown conflicting results [6,26,35]. Also, the HIV studies mentioned focused on prenatal or intra-partum transmission, whereas our study modeled postnatal HIV transmission via breastfeeding by oral inoculation of 1-month old infant macaques with SIVmac251-5/98. The route(s) of infection in utero or during birth for individual infants and source of virus (cell-free or cell-associated) is usually unknown, and therefore different mechanisms may be responsible for viral transmission via these routes [6]. Consistent with this view, others have reported that more SIV variants were detected in orally infected newborn macaques than in infants born to SIV-infected female macaques for which transmission occurred in utero [36] or during the late breast-feeding period [37].

Neither of the SIV vaccines used in this experiment (MVA-SIVgpe and SIVmac1A11), nor the presence of maternal antibodies in one of the MVA-SIVgpe immunized groups altered which envelope variants were transmitted because in each group, some monkeys became infected with more heterogeneous and others with more homogeneous virus populations. It is possible that neither MVA-SIVgpe nor SIVmac1A11 elicited immune responses that effectively targeted the predominant SIV *env *variants in the SIVmac251-5/98 stock, or that anti-envelope immune responses were elicited against regions of the envelope other than V1–V2. It is also possible that vaccine-induced immune mechanisms at the time and/or site(s) of initial infection were not potent enough to modulate the variant transmission patterns.

Viral levels in plasma of monkeys with more homogeneous populations of SIV *env *variants tended to be lower one week after oral inoculation with SIVmac251-5/98. The higher initial virus levels in infants infected with multiple variants may reflect higher replication capacities of diverse variant populations compared to those comprised of one main variant, especially in the initial target cells during the first days of infection. We have observed this previously for adult macaques inoculated intravaginally [22]. From the second week after SIVmac251-5/98 inoculation onwards, however, there was no correlation between viral genetic complexity (measured by entropy) or divergence (measured by MMS) and plasma SIV RNA levels. Thus, once systemic infection was established, virus replication attained similar levels regardless of the initial diversity, and there was no difference in AIDS-free survival times.

Based on the measurement of MMS values, we observed little change or a decrease in genetic divergence of plasma SIV *env *variant quasispecies in all unvaccinated and most vaccinated animals that developed AIDS within the observation period of 28 weeks. In contrast, diversification in plasma SIV envelope variant populations was observed in 4 of the 8 vaccinated monkeys that were still relatively healthy at 28 weeks. This increased divergence of plasma viral variants at ~3 to 5 months after infection was generally associated with more sustained levels of SIV-specific neutralizing antibodies, and also of SIV Gag and Env-specific antibodies (measured by ELISA, as shown previously [21]). Similar associations between viral genetic divergence, immune parameters and/or disease progression have been described in HIV-infected adults and children [6,8,12,13,15,16,29,38-44], and recently also in juvenile macaques following intravenous or intra-rectal SIVsm inoculation [45]. Our studies extend these observations by demonstrating that this correlation of more sustained immune responses, enhanced viral divergence and slower disease progression is also observed in infant macaques following oral SIV infection. Together, these results suggest that the rate of virus evolution is determined by a combination of the extent of virus replication (which induces random mutations due to the error-prone reverse transcriptase) and selection pressures such as antiviral immune responses that promote the outgrowth of new variants. The generation of increasingly divergent viral variants ("immune escape mutants") reflects attempts of the immune system, albeit only partially effective, to control virus replication. In contrast, high viremia and little evolution of viral envelope variants is associated with severe immunodeficiency (and thus little immune selection pressure) and rapid disease progression.

## Conclusions

The patterns of SIV *env *variant transmission and evolution in infant macaques that were inoculated orally with the same SIVmac251-5/98 stock reflect the range of results that is observed in mother-to-infant transmission of HIV, where the dose and genetic diversity of the virus at the time of transmission are unknown. While the vaccines tested here did not modulate oral transmission of viral variants, an association was found between vaccination and enhanced antiviral immune responses, increased *env *diversity, and a slower disease course. These findings are similar to observations in HIV-infected children with slow disease progression and underscore the relevance of the infant macaque model for developing neonatal vaccine strategies to prevent pediatric HIV infection and AIDS [46]. These results also support the concept that neonatal immunization could prevent rapid disease progression in infants who become HIV-infected by breast-feeding.

## Materials and Methods

### Infant immunizations, virus inoculations, and sample collection

All newborn rhesus macaques (*Macaca mulatta*) were from the HIV-2, SIV, type D retrovirus, and simian T-cell lymphotropic virus type 1-free colony at the California National Primate Research Center. Newborn monkeys were hand-reared in a primate nursery, and all animals were housed in accordance with American Association for Accreditation of Laboratory Animal Care standards. We adhered to the "Guide for Care and Use of Laboratory Animals" [47]. When necessary, animals were immobilized with 10 mg/kg ketamine hydrochloride (Parke-Davis, Morris Plains, NJ) injected intramuscularly (IM). EDTA-anticoagulated blood samples were collected regularly for monitoring virologic and immunologic parameters as described previously [21].

Four newborn macaques had maternally derived SIV antibodies, because their mothers had been immunized and boosted during three or four consecutive pregnancies with whole-inactivated SIVmac251 plus Montanide ISA 51 adjuvant (Seppic, Fairfield, NJ), administered intramuscularly as previously described [21]. One of two SIV vaccines was administered to newborn monkeys at birth and 3 weeks of age: Modified Vaccinia virus Ankara expressing SIVmac239 gag, pol, and env (MVA-SIVgpe) was given to 8 newborn monkeys, including the 4 with maternal antibodies. SIVmac1A11 was given to 4 newborn monkeys. Details about these vaccines are described elsewhere [21].

At 4 weeks of age, these 17 monkeys were inoculated orally with 2 doses (24 hours apart) of uncloned virulent SIVmac251. Ketamine anesthesia was used for each inoculation. Each dose consisted of 1 ml of undiluted SIVmac251 of a stock designated by lot number -5/98, and was administered atraumatically by dispensing virus slowly into the mouth with a syringe. The SIVmac251-5/98 virus stock used in this study was derived from a previous SIVmac251 stock (lot 8/95) that was serially passaged intravenously in rhesus macaques as described [21]. This SIVmac251-5/98 stock contained 1 × 10^5 ^50% tissue culture infective doses (TCID_50_) and 1.4 × 10^9 ^copies of RNA per ml (determined by bDNA assay).

### Quantitation of plasma viral RNA

Viral RNA in plasma was quantified using a branched DNA (bDNA) signal amplification assay specific for SIV, with conditions as described previously [22].

### RNA isolation and RT-PCR

RNA was extracted from plasma samples (100–140 μl) using a viral RNA isolation kit (Qiagen, Inc., Valencia, CA) following the manufacturer's protocol. A 590 bp fragment encompassing the V1–V2 region of SIV *env *was then amplified in a nested RT-PCR assay as previously described [22].

### Analysis of SIV variants by heteroduplex mobility assay (HMA)

Genetic diversity in viral variant populations was analyzed using a modification of the HMA methods described elsewhere [22,48]. In brief, V1–V2 *env *fragments were generated by RT-PCR as described above, and the presence of sufficient product was confirmed on a 1.5% agarose gel. The RT-PCR products were then mixed with 1.5 μl of 10× annealing buffer (1 M NaCl, 100 mM Tris, 20 mM EDTA), denatured at 94°C for 2 minutes and placed immediately on wet ice to promote heteroduplex formation. Samples were then run on non-denaturing 5% polyacrylamide gels and stained with ethidium bromide (0.5 μg/ml). Reverse-images of the stained gels were photographed with a digital imaging system (Alpha Innotech Corporation, San Leandro, CA). All gel images were color reversed to enhance visualization of banding patterns (e.g. black bands on white background). The number of heteroduplex bands observed is a measure of SIV envelope diversity in each monkey's virus population (i.e. a large number of bands on a gel corresponds to a large number of V1–V2 variants in the sample). The RT-PCR and HMA analysis on plasma samples was performed in replicates (of at least 2) to assure reproducibility of the gel banding patterns.

To further characterize SIV envelope variants, we used an additional form of HMA analysis that assesses the relative genetic similarity of specific viral variants by comparing the HMA patterns that result from combinations of these variants. Mixtures of SIV RNA from plasma samples were analyzed by HMA to allow estimation of sequence similarity between two different homogeneous virus populations (i.e. the most common inoculum variants and/or plasma variants from infected monkeys). This "mixture analysis" is based on the HMA HIV subtyping protocol developed by Delwart et al. [49] and was performed as described previously [22]. Briefly, mixtures of equal volumes of SIV V1–V2 *env *PCR product amplified from two different samples were mixed and subjected to HMA analysis as described above. Mixtures that result in a single homoduplex band are 98–100% identical [48] in the nucleotide sequence of the PCR fragment analyzed (V1–V2 *env *region). Mixtures that result in the formation of heteroduplexes are comprised of variant populations with nucleotide sequences that differ by more than 1–2% or have an insertion/deletion (i.e., a single codon length difference will also cause a gel shift) [48]. We have validated this HMA method for SIVmac251 in a previous study [22].

### Calculation of entropy and median mobility shift

All measures of entropy (E) and median mobility shift (MMS) were estimated according to methods described by Delwart et al. [8]. Images from HMA gels were captured with a CCD camera as binary TIFF files and color reversed to enhance visualization of banding patterns (e.g. black bands on white background). Each TIFF gel image file was then opened using Adobe Photoshop Version 6.0 (Adobe Inc., San Jose, CA) and edited to ensure that the lightest inter-lane areas of the gel image had "0" signal intensity (as read by the NIH Image Program and required for the Hdent program described below). Digitized gel lanes were scanned by using the plot profile function of the NIH Image Program (available at ). Lane scans within the same gel were of equal length (i.e. same number of pixels) and were recorded from positions immediately below the single-stranded DNA position to immediately below the homoduplex. The signal intensity at each pixel along the scan was transferred to a Microsoft Excel (Richmond, Wash.) file. Because different numbers of pixels per lane were acquired from different gels, each gel was standardized by partitioning into 191 divisions, the smallest number of pixels in the scans under study. This allowed the maximum distinction of fine banding patterns, while permitting unbiased comparison between gels.

The quasispecies diversity for each sample was estimated by calculating a normalized Shannon entropy, a measure of the breadth or spread of the signal distribution in each HMA gel lane, using the HDent program (available at ) as described by Delwart et al. [8]. The Shannon entropy (*S*) is defined as: *S *= - Σ (from *i *= 1 to *N*)*P*(i)ln [*P*(i)], where *N *is the number of partitions in a lane, and *P*(i) is the fraction of the total signal in partition *i*. The maximum possible entropy is ln(*N*), and we defined the normalized entropy as S/ln(*N*). The normalized entropy has a range of 0 to 1, where 0 reflects no diversity (all of the signal is in a single partition), and 1 reflects maximum entropy, in which the signal is evenly distributed throughout all partitions in the lane. Thus, entropy is large for lanes with many, closely spaced or overlapping bands and small for lanes with only one band or a few, narrow bands.

Shannon entropy estimates quasispecies genetic diversity by measuring the pattern of SIV V1–V2 *env *heteroduplex distribution in an HMA gel lane rather than the specific electrophoretic mobility of heteroduplexes. However, the electrophoretic mobility of heteroduplexes through a polyacrylamide gel is proportional to the sequence differences in reannealed DNA strands [38,48,49]. The degree of SIV quasispecies envelope sequence divergence among the V1–V2 *env *variants present in plasma samples was estimated by calculating a median mobility shift (MMS) for each HMA gel lane using the HDent program. The MMS is a measure of the midpoint of the total signal in an HMA gel lane that has values between 0 and 1, where 0 corresponds to the bottom of an HMA gel lane (i.e. nearest homoduplex bands) and 1 corresponds to the top of the lane. Thus, a MMS score of 1 reflects maximum sequence diversity (i.e. all heteroduplexes bands have maximum mobility reduction and no visible homoduplexes); a MMS value of 0 reflects maximum sequence similarity (> 98%) where all signal for a lane is in homoduplex bands and there are no visible heteroduplexes.

### Assessment of MHC class I alleles

DNA extracted from lymphoid cells (with QIAamp^® ^DNA mini kit, QIAgen, Valencia, CA) was used to screen for the presence of the rhesus macaque major histocompatibility complex (MHC) class I alleles Mamu A*01 and Mamu B*01, using a PCR-based technique [50,51].

### Neutralizing antibodies

Neutralizing antibody titers in EDTA-anticoagulated plasma were measured according to methods described previously [52], except that CEM-R5 cells (i.e. CEM×174 cells expressing CCR5 by transfection; generously provided by James Robinson) were used. Neutralizing antibody titers were expressed as the reciprocal of the plasma dilution at which 50% of cells were protected from virus-induced killing as measured by neutral red uptake. The virus consisted of SIVmac251-5/98 briefly propagated in human PBMC.

### Statistical analysis

Fisher's exact test, performed with Instat v. 2.03 (GraphPad Software, Inc., San Diego, CA), was used to evaluate possible association of SIV *env *V1–V2 variants detected by HMA in plasma of monkeys one week after oral SIVmac251 inoculation with the levels of plasma viral RNA at this same time point. To determine potential linear associations of Entropy values or MMS values over time and SIV neutralizing antibody levels over time, linear regression was performed using Prism v.3.0 (GraphPad Software Inc., San Diego CA). For all statistical comparisons, a P value less than 0.05 was considered significant.

## Competing interests

The author(s) declare they have no competing interests.

## Authors' contributions

JG carried out the HMA studies and drafted the manuscript; KVR participated in the design and coordination of the study, acquisition and analysis of data, and helped draft the manuscript; DM analyzed and interpreted all neutralizing antibody assays; PE and BM designed and provided the MVA constructs, participated in the experimental design and manuscript writing; MM designed and coordinated the study, assisted in the data analyses and helped draft the manuscript.
